# Supplementary material for: Effect of belt electrode-skeletal muscle electrical stimulation on immobilization-induced muscle fibrosis
Source: PLoS One. 2021 May 13;16(5):e0244120. doi: 10.1371/journal.pone.0244120 (PMC8118259; doi:10.1371/journal.pone.0244120)
Supplement: Attachment — Submitted filename: Response to Reviewers.docx [file pone.0244120.s002.docx]

Response to the Editor-in-Chief and reviewer

We would like to thank both the reviewers for the evaluation of our study, as well as their constructive criticism, which allowed us to strengthen and clarify our study's conclusions. We have responded in detail to each comment and discussed how the concerns raised were addressed in the revised manuscript.

Journal Requirements

***1. Please ensure that your manuscript meets PLOS ONE's style requirements, including those for file naming. The PLOS ONE style templates can be found at https://journals.plos.org/plosone/s/file?id=wjVg/PLOSOne_formatting_sample_main_body.pdf and https://journals.plos.org/plosone/s/file?id=ba62/PLOSOne_formatting_sample _title_authors_affiliations.pdf***

We rechecked our manuscript according to The PLOS ONE style templates.

***2.1. Please provide an amended Funding Statement declaring this commercial affiliation, as well as a statement regarding the Role of Funders in your study. If the funding organization did not play a role in the study design, data collection and analysis, decision to publish, or preparation of the manuscript and only provided financial support in the form of authors' salaries and/or research materials, please review your statements relating to the author contributions, and ensure you have specifically and accurately indicated the role(s) that these authors had in your study. You can update author roles in the Author Contributions section of the online submission form.***

We updated author roles of “Atsushi Nawata” and “Ryuji Akimoto” to “Resources”. Additionally, please add following statement to our amended Funding Statement:

“The funder provided support in the form of salaries for Akimoto R and Nawata A, but did not have any additional role in the study design, data collection and analysis, decision to publish, or preparation of the manuscript. The specific roles of these authors are articulated in the ‘author contributions’ section.”

***2.2. Please also provide an updated Competing Interests Statement declaring this commercial affiliation along with any other relevant declarations relating to employment, consultancy, patents, products in development, or marketed products, etc.***

We confirmed that this commercial affiliation does not alter our adherence to all PLOS ONE policies on sharing data and materials by including the following. Please add following statement to our Competing Interests Statement:

“This does not alter our adherence to PLOS ONE policies on sharing data and materials.”

Reviewer: 1

***The study could benefit from having a control groups for B-SES.***

Your advice is very important. However, the Ethics Review Committee for Animal Experimentation of Nagasaki University warned this study to use the minimum number of rats required. Therefore, we could not add the control + B-SES group in this research. Presently, we are considering this point in another project.

***Please list the ages of the rats***

We used “Eight-week-old male Wistar rats” (page 6, line 109).

***Please list whether rats were individually, or group housed.***

Two rats were housed in each cage (page 6, line 111).

***Please list the reagents/kits used to perform the stains. Or at the very least, reference a paper indicating the use of those stains in your lab or other***

We added the reagents/kits used to each staining and references related to stain protocols (page 9, line 172-176). Also, we added following research in reference section (page 21, line 457-459).

14. Hintz CS, Coyle EF, Kaiser KK, Chi MM, Lowry OH. Comparison of muscle fiber typing by quantitative enzyme assays and by myosin ATPase staining. J Histochem Cytochem 1984 ;32: 655-660.

***Please indicate whether primer pairs were validated with the housekeeping gene (e.g., did gene of interest and housekeeping gene amplify targets with similar efficiency).***

We confirmed amplification efficiency of all primer pairs in pilot study. In addition, the amplification efficiency of all primer pairs was 90-110%. From these data, we considered that the gene of interest and housekeeping gene amplified targets with similar efficiency.

***Please indicate*** ***the amount of cDNA used for amplification.***

The cDNA concentration of all samples was unified to 25 ng/μl. And, we applied 0.2 μl cDNA to each well.

***Please indicate whether*** ***PCR products were confirmed to be specific by sequencing.***

We could not research the PCR products by the sequencer device. However, we confirmed the specification of PCR products with Basic Local Alignment Search Tool (BLAST, NIH, US). All PCR products were indicated high percent identify and low E-value, no sequence of molecule different from the target was observed.

***Please define ROM***

We described definition of ROM according your advice (page 8, line 157-162).

***Figure 2 suggests that the density of myonuclei is greater in BES group vs immobilization or control (same number of nuclei compared to control, but decreased area; greater number of nuclei compared to immobilization but same area as immobilization). This suggest that BES is decreasing atrophy of the muscle fibers. However, in Figure 5, two markers of atrophy are not decreased by BES. Can the authors please comment in the discussion regarding this observation and how they are compatible with one another?***

Thank you for your important suggestion. The number of myonuclei was significantly higher in the B-SES group than in the immobilization group, whereas there was no significant difference between the B-SES and control groups. However, the myonuclear domain size, which is defined as the cytoplasmic region controlled by a single myonuclei, and Atrogin-1/MuRF-1 mRNA expression in Immobilization and B-SES groups were no significant difference. In summary, the muscle contractile exercise through B-SES may prevent the decreasing myonuclei, but not may be possible to suppress the decrease in muscular protein produced by single myonuclei.

Myofiber atrophy was caused by the loss of myonuclei and the upregulation of degrading system of muscular protein. From our results, the muscle contractile exercise through B-SES had no effect for mitigating the upregulation of degrading system of muscular protein, despite the preventing the decrease in myonuclei. Therefore, we surmised that mitigation of decreasing myonuclei via B-SES led to suppress the partial type I myofiber atrophy. Therefore, we revised sentence according the above contents (page 17, line 372- page 18, line 378).

***For Figure 6C, please provide rationale for measuring 100 fibers per group. For example, provide a reference or rationale indicating that it either the standard or allows for adequate sampling.***

Our laboratory presented the data by this method in previous works (following articles). Therefore, we added reference 3 and 13 to page 9, line 183.

3. Honda Y, Sakamoto J, Nakano J, Kataoka H, Sasabe R, Goto K, et al. Upregulation of interleukin-1beta/transforming growth factor-beta1 and hypoxia relate to molecular mechanisms underlying immobilization-induced muscle contracture. Muscle Nerve 2015;52: 419–427.
13. Matsumoto Y, Nakano J, Oga S, Kataoka H, Honda Y, Sakamoto J, et al. The non-thermal effects of pulsed ultrasound irradiation on the development of disuse muscle atrophy in rat gastrocnemius muscle. Ultrasound Med Biol 2014;40: 1578-1586.

***Some studies suggest that macrophages are important for promoting recovery from atrophy. Please include a few sentences or paragraph concerning how the current findings fit within data suggesting a protective role of macrophages.***

We added new sentences according your suggestion (page 18, line 381-385).

***Lines 397-400, page 18, please check this sentence as it is can be improved on clarity. “Namely, the macrophages accumulation via the myonuclei decrease was suppressed by muscle contractile exercise through B-SES, our investigation surmised that this alteration led to the prevention of immobilization-induced muscle fibrosis via the suppression of fibrosis related factor”***

We revised this sentence according your suggestion (page 18, line 397- page 19, line 401).
