# Supplementary material for: Effect of belt electrode-skeletal muscle electrical stimulation on immobilization-induced muscle fibrosis
Source: PLoS One. 2021 May 13;16(5):e0244120. doi: 10.1371/journal.pone.0244120 (PMC8118259; doi:10.1371/journal.pone.0244120)
Supplement: Attachment — Submitted filename: Response to Reviewers.docx [file pone.0244120.s003.docx]

Response to reviewer

We would like to thank the reviewer for the evaluation of our study, as well as constructive criticism, which allowed us to strengthen and clarify our study's conclusions. We have responded in detail to each comment and discussed how the concerns raised were addressed in the revised manuscript.

Reviewer:

***I recommend stating what you did in your response. So eliminate the highlighted lines 372-378 on the redlined manuscript and replace with below (your response).*** ***“Myofiber atrophy was caused by the loss of myonuclei and the upregulation of degrading system of muscular protein. From our results, the muscle contractile exercise through B-SES had no effect for mitigating the upregulation of degrading system of muscular protein, despite the preventing the decrease in myonuclei. Therefore, we surmised that mitigation of decreasing myonuclei via B-SES led to suppress the partial type I myofiber atrophy”***

Following your advice, we replaced this sentence to your recommendation (page 17-18, line 374-379).

***The paragraphs regarding macrophages do not address the point raised (page 18, lines 381- 385). If macrophages may be beneficial, then B-SES might not be a good long-term for preventing muscle atrophy. Meaning, you are preventing macrophage accumulation, yet some studies suggest that macrophages are important for preventing atrophy. This was what I wanted you to address.***

We apologized for wronging the mean of your question.

Macrophages were divided into M1 macrophages (inflammatory macrophages) and M2 macrophages (anti-inflammatory macrophages). M1 macrophages related to the proliferation of myoblasts, M2 macrophages matured myoblasts to myotube. Therefore, some researches with reload animal models indicated that M1 macrophages accumulation was beneficial for muscle hypertrophy. However, some researches for fibrous lesions demonstrated that M1 macrophages played a key role of fibrosis via IL-1β/TGF-β1 signaling.

In our previous study, although M1 macrophages increased in 2-week immobilized rat skeletal muscles (Oga S, Honda Y, et al. Muscle Nerve 61(5): 662-670, 2020), the muscle atrophy occurred in same muscles. In this study, M2 macrophages did not increase even though M1 macrophages increased. We surmised that M1 macrophages did not work for preventing muscle atrophy in this immobilization model. On the other hand, immobilization-induced muscle fibrosis occurred in same immobilized rat skeletal muscles, Additionally, the expression of IL-1β/TGF-β1 mRNA were upregulated (unpublished data). Therefore, M1 macrophages might relate to promote immobilization-induced muscle fibrosis, we surmised that the inhibition of M1 macrophages accumulation via B-SES led to prevent immobilization-induced muscle fibrosis.

Since this investigation was examined for the effect of B-SES to immobilization-induced muscle fibrosis, we added the above content about fibrosis to “Discussion” (page 18, line 381-388). And, we added following reports to “References” (page 23, line 502-503).

28.　Oga S, Goto K, Sakamoto J, Honda Y, Sasaki R, Ishikawa K, et al. Mechanisms underlying immobilization-induced muscle pain in rats. Muscle Nerve 2020;61: 662-670.

***Please include in the methods the amount of cDNA added to each well. Although the authors stated it in the response, which I appreciate, the point was to include that information in the manuscript so that others can better reproduce the data.***

We described the amount of cDNA added to each well according your advice (page 11, line 216-217).
